# Highly Efficient and Selective Hydrogenation of Biomass-Derived Furfural Using Interface-Active Rice Husk-Based Porous Carbon-Supported NiCu Alloy Catalysts

**DOI:** 10.3390/molecules29112638

**Published:** 2024-06-03

**Authors:** Zhiyao Ding, Yujun Gao, Lianghai Hu, Xiaomin Yang

**Affiliations:** 1 School of Life Sciences, Jilin University, Changchun 130012, China; dingzy17@mails.jlu.edu.cn (Z.D.); yjgao18@mails.jlu.edu.cn (Y.G.); 2 College of Chemistry, Jilin University, Changchun 130012, China

**Keywords:** NiCu bimetallic catalyst, furfural, tetrahydrofurfuryl alcohol

## Abstract

A series of bimetallic Ni_x_Cu_y_ catalysts with different metal molar ratios, supported on nitric acid modified rice husk-based porous carbon (RHPC), were prepared using a simple impregnation method for the liquid-phase hydrogenation of furfural (FFA) to tetrahydrofurfuryl alcohol (THFA). The Ni_2_Cu_1_/RHPC catalyst, with an average metal particle size of 9.3 nm, exhibits excellent catalytic performance for the selective hydrogenation of FFA to THFA. The 100% conversion of FFA and the 99% selectivity to THFA were obtained under mild reaction conditions (50 °C, 1 MPa, 1 h), using water as a green reaction solvent. The synergistic effect of NiCu alloy ensures the high catalytic activity. The acid sites and oxygen-containing functional groups on the surface of the modified RHPC can enhance the selectivity of THFA. The Ni_2_Cu_1_/RHPC catalyst offers good cyclability and regenerability. The current work proposes a simple method for preparing an NiCu bimetallic catalyst. The catalyst exhibits excellent performance in the catalytic hydrogenation of furfural to tetrahydrofurfuryl alcohol, which broadens the application of non-noble metal bimetallic nanocatalysts in the catalytic hydrogenation of furfural.

## 1. Introduction

Non-precious metal Ni catalysts have been widely studied for the hydrogenation of furfural to produce valuable chemicals such as tetrahydrofurfuryl alcohol and furfuryl alcohol (FOL) [1,2,3,4]. In contrast, non-precious metal bimetallic NiCu catalysts exhibit very high catalytic activity and selectivity due to their tunable composition, controlled particle dispersion, and synergistic effects between different metals [5,6,7]. Seemala et al. [8] synthesized Cu-Ni/TiO_2_ catalyst to convert HMF and furfural into methyl furan, and the Cu-Ni/TiO_2_ catalyst showed better stability and regenerability (reaction conditions: T = 200 °C, p (H_2_) = 2.5 MPa). Wang et al. [9] synthesized CuNi@C catalyst by embedding CuNi bimetallic nanoparticles into a carbon matrix and applied it to the selective hydrogenation of furfural to cyclopentanone. The metallic copper and nickel particles have a nanometer size of about 15 nm. The CuNi_0.5_@C catalyst showed the best catalytic performance under optimal conditions (130 °C, 5 h, 5 MPa) (the furfural conversion was 99.3%; the cyclopentanone yield was 96.9%). In addition, the CuNi_0.5_@C catalyst can be reused four times, with good activity and stability. Liu et al. [10] synthesized bimetallic Cu-Ni/CNTs catalysts exhibiting 100% conversion to furfural and up to 90.3% selectivity to THFA at 130 °C, 40 bar hydrogen pressure, and 10 h of reaction. Wu et al. [11] prepared Cu_x_Ni_y_/MgAlO (x/y = 7:1, 3:1, 1:1, 1:3, 1:7) catalysts with different molar ratios under optimal reaction conditions (150 °C, 4 MPa, 3 h). The furfural can be selectively converted to furfuryl alcohol or tetrahydrofurfuryl alcohol (the selectivity of FOL is >99%, and the selectivity of THFA is 95%). However, the above-mentioned NiCu catalyst still requires a higher temperature or pressure during the catalytic hydrogenation of furfural [12]. Therefore, for the furfural hydrogenation reaction, it is more economical and attractive to develop a non-precious metal bimetallic nanocatalyst that is efficient, stable, and environmentally friendly under mild conditions.

Carbon materials (AC, CB, CNTs) exhibit a high specific surface area, a developed pore space, and surface-controllable chemical properties. These characteristics can affect catalyst activity and product selectivity [13]. According to reports, after the activated carbon is treated with nitric acid, a variety of oxygen-containing functional groups (such as carboxyl, lactone, and phenolic hydroxyl groups) will be introduced on the surface [14]. The presence of these oxygen-containing functional groups is beneficial to the dispersion and selectivity of metal nanoparticles [15,16]. In this work, a nitric acid modified surface using low-cost, environmentally-friendly rice husk-based porous carbon (RHPC) will increase the number of oxidative functional groups and acid sites [17]. The modified RHPC was used as a support, and Ni_x_Cu_y_/RHPC catalysts with different Ni/Cu ratios were synthesized by impregnation for the catalytic hydrogenation of furfural. The results show that the Ni_2_Cu_1_/RHPC catalyst can efficiently convert FFA to THFA (the FFA conversion rate is 100%, the THFA selectivity is >99%, and the carbon balance is 97%), under mild conditions (50 °C, 1 MPa, 1 h). The preparation of bimetallic CuNi catalysts in this work is inexpensive and simple, which makes them promising candidates for the conversion of furfural to THFA.

## 2. Results and Discussion

### 2.1. Characterization of Catalysts

**Figure 1:**
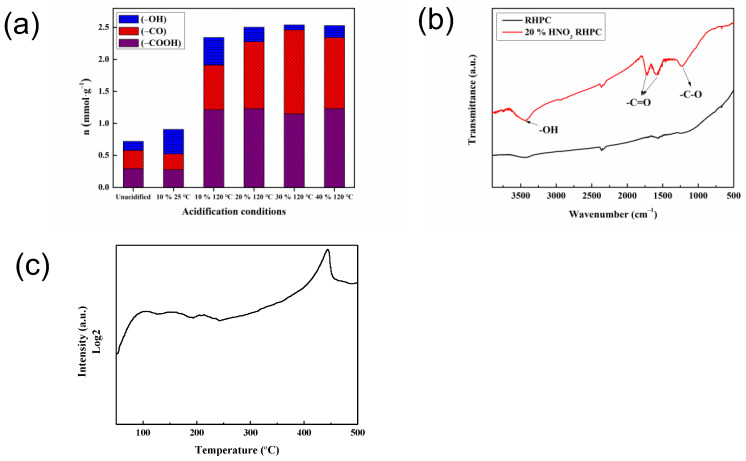
(**a**) Boehm titration results of acid functional groups on the RHPC surface. (**b**) FT-IR spectra of RHPC and 20% HNO_3_ RHPC. (**c**) NH_3_-TPD result of Ni_2_Cu_1_/RHPC catalyst.

The acidic functional Boehm titration results on the surface of RHPC after different acidification conditions are shown in Figure 1a. It can be seen from the figure that the acidic functional groups on the RHPC changed very significantly after HNO_3_ treatment. When acidified with 10% HNO_3_ at room temperature, the total acid content on the RHPC is 0.91 mmol/g, but the total acid content of RHPC without acidification is 0.72 mmol/g; thus, the total acid content increased slightly. When using 10% HNO_3_ at 120 °C, the total acid content is 2.34 mmol/g; it can be seen that the acidic functional groups of RHPC increase significantly under heating conditions, which indicates that heating contributes to the formation of acidic functional groups. When the nitric acid concentration was between 20% and 40%, the growth of the acidic functional groups on RHPC tends to be gentle; therefore, we chose 20% HNO_3_ acidification as the optimal condition.

The FT-IR results of RHPC, before and after 20% HNO_3_ acidification treatment, are shown in Figure 1b. It can be seen from the figure that after acidification, the RHPC exhibits a very obvious peak change, i.e., a −OH absorption peak at 3421 cm^−1^, and −C=O and −CO absorption at 1734 cm^−1^ and 1228 cm^−1^, respectively, and these results are consistent with those in the literature [15]. As can be seen from the figure, the acidified RHPC expressed a very obvious peak change, with −OH absorption peak at 3421 cm^−1^, and −C=O and −C-O absorption peaks at 1734 cm^−1^ and 1228 cm^−1^, respectively, which are consistent with these results in a previous report. This indicated the presence of hydroxyl, carboxyl, and carbonyl functional groups on the surface of RHPC modified with 20% HNO_3_, which is in agreement with the Boehm titration results. The increase in the surface density of the oxygen-containing groups on the surface of rice husk-based porous carbon after nitric acid modification helps to augment the polarity and wettability of the samples. The NH_3_-TPD results for the Ni_2_Cu_1_/RHPC catalyst are shown in Figure 1c. The first peak (100–300 °C) in the figure should be assigned to the weakly acidic site. The two peaks (300–500 °C) corresponded to the position of the moderate acid. Therefore, there were acid sites on the surface of the prepared Ni_2_Cu_1_/RHPC catalyst. The increased acidic sites on the RHPC treated with nitric acid can promote the activation of furfuryloxy intermediates, and furfuryloxy intermediates, as active adsorbates, play an important role in the formation of FOL and THFA [16].

The composition of Ni and Cu in the Ni_x_Cu_y_/RHPC catalysts is shown in Table 1. The concentration of Ni and Cu inNi_x_Cu_y_/RHPC nanocatalysts with different Ni/Cu mole ratios were characterized by ICP-AES, as shown in Table 1. It can be seen that the real and theoretical bulk Ni/Cu ratio values were quite similar.

**Figure 2:**
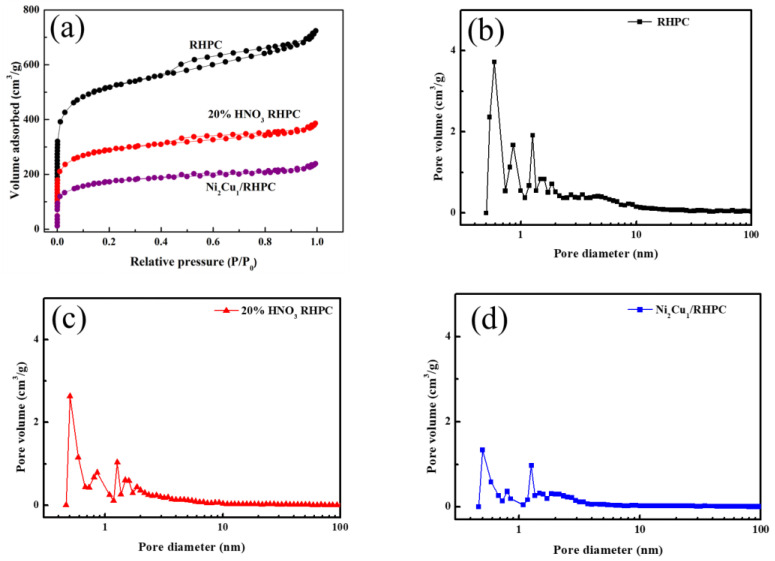
(**a**) N_2_ adsorption–desorption isotherms and pore size distribution curves of (**b**) PHPC, (**c**) pf 20% HNO_3_ RHPC, and (**d**) Ni_2_Cu_1_/RHPC samples.

The nitrogen adsorption and desorption characteristics of the RHPC and Ni_2_Cu_1_/RHPC catalysts, before and after nitric acid treatment, are shown in Figure 2a. It can be seen from the figure that the curve is a typical I-type isotherm and H4 hysteresis loop. When P/P_0_ < 0.1 in the low-pressure region, as the P/P_0_ value increased, the curve sharply rose, which showed that the material also has a rich microporous structure. When the results are within this range, this indicates that the catalyst material contains mesopores. The pore size distribution curves of RHPC are shown in Figure 2b–d, and the average pore diameter is shown in Table 2, as statistical table of pore size and pore volume of RHPC and Ni_2_Cu_1_/RHPC catalysts, before and after nitric acid treatment. It can be seen from the table that the specific surface area and the volume of micropores and mesopores of the porous carbon of rice husks become smaller after acid treatment. This is because part of the pores may collapse during the formation of surface groups by the nitric acid oxidation of rice husk-based porous carbon, which is consistent with the results in the literature [18]. At the same time, during the high-temperature reduction process of the catalyst, some of the pores will collapse, which will cause the specific surface area to decrease [19].

**Figure 3:**
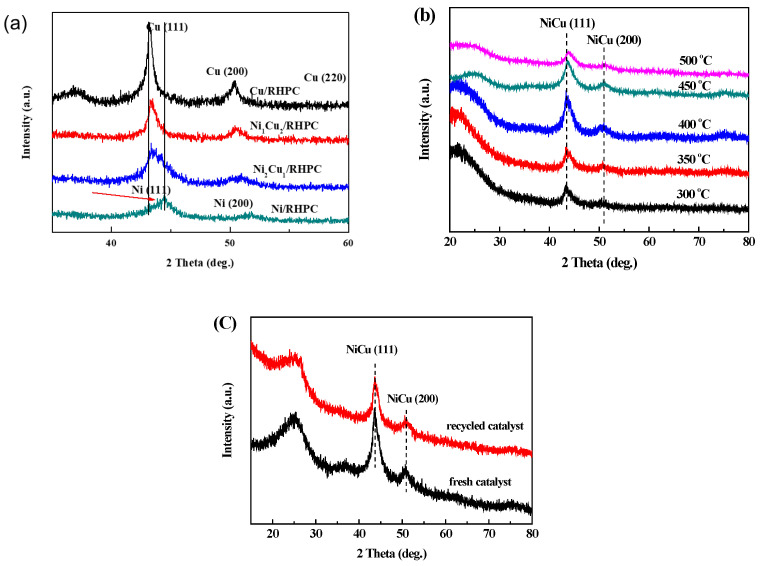
XRD patterns of (**a**) Ni_x_Cu_y_/RHPC catalysts with different Ni/Cu molar ratios, (**b**) Ni_2_Cu_1_/RHPC catalysts with different reduction temperatures, and (**c**) fresh and recycled Ni_2_Cu_1_/RHPC catalysts.

The XRD patterns of Ni_x_Cu_y_/RHPC catalysts with different Ni/Cu molar ratios are displayed in Figure 3a. The XRD pattern presented characteristic peaks at 2θ = 43.2°, 50.4°, and 74.1° for Cu/RHPC catalysts, which could be indexed to Cu (111), Cu (200), and Cu (220) lattice planes [9]. At the same time, the Ni/RHPC catalyst showed two diffraction peaks at 2θ = 44.5° and 51.8°, assigned to the Ni (111) and Ni (200) crystal planes, respectively. Interestingly, the diffraction peaks of Ni_1_Cu_2_/RHPC and Ni_2_Cu_1_/RHPC appeared between the Cu (111) and Ni (111) reflections. The existence of diffraction peaks between the Cu (111) and Ni (111) reflections for the bimetallic catalysts are evidence of the formation of NiCu alloy phases [8,20,21].

In order to investigate the effect of reduction temperature on the structure of bimetallic NiCu nanocatalysts, we conducted systematic activation experiments on Ni_2_Cu_1_/RHPC within broad temperature ranges from 300 °C to 500 °C. Under precisely controlled experimental conditions, it was observed that after the catalyst underwent reduction treatments at 300 °C and 500 °C, it exhibited two distinct characteristic diffraction peaks in the X-ray diffraction (XRD) patterns. Specifically, the diffraction peak located at 2θ = 43.3–43.5° corresponds to the (111) crystal plane of the NiCu alloy, while the peak at 2θ = 50.5–51.0° belongs to the (200) crystal plane. These precise diffraction data provided us with crucial information regarding the crystal plane structure of the catalyst. However, it is noteworthy that when the reduction temperature increased to 500 °C, the diffraction peak intensity of Ni2Cu1/RHPC significantly decreased, reflecting its poor crystallinity. The XRD pattern of the Ni_2_Cu_1_/RHPC catalyst, before and after the cycle, is shown in Figure 3c. It can be seen from the figure that the peak height of the diffraction peak of the catalyst slightly decreases after the reaction. This indicates that the crystallinity of the catalyst slightly decreased after the reaction.

**Figure 4:**
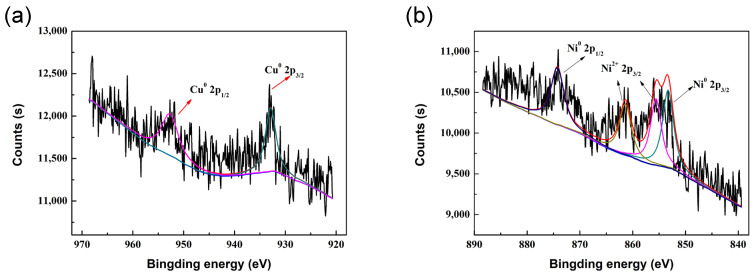
XPS spectra of Ni_2_Cu_1_/RHPC catalyst. (**a**) The XPS spectrum of Cu 2p. (**b**) The XPS spectrum of Ni 2p.

The Cu 2p and Ni 2p peaks in the XPS spectrum of the Ni_2_Cu_1_/RHPC catalysts were used to determine the oxidation state and surface composition of the bimetallic catalyst. The XPS spectrum of Cu 2p is shown in Figure 4a. The binding energy of Cu2p_3/2_ is 932.9 eV, and the binding energy of Cu2p_1/2_ is 952.7 eV, which can be attributed to Cu^0^ [9]. The XPS spectrum of Ni 2p is shown in Figure 4b. The binding energy of Ni2p_3/2_ is 853.3 eV, and the binding energy of Ni2p_1/2_ is 874.26, which was attributed to Ni^0^ [22,23,24]. The peaks at 855.6 eV, 861.4 eV, and 871.8 eV were attributable to NiO [25,26]. This indicates that Ni^0^ and Cu^0^ are present, and an NiCu alloy is formed. Together, the XRD and XPS results illustrate the formation of an NiCu alloy. At the same time, the XPS pattern shows the presence of Ni^2+^, which can be attributed to the partial oxidation of Ni^0^ in the environment [8,27,28].

**Figure 5:**
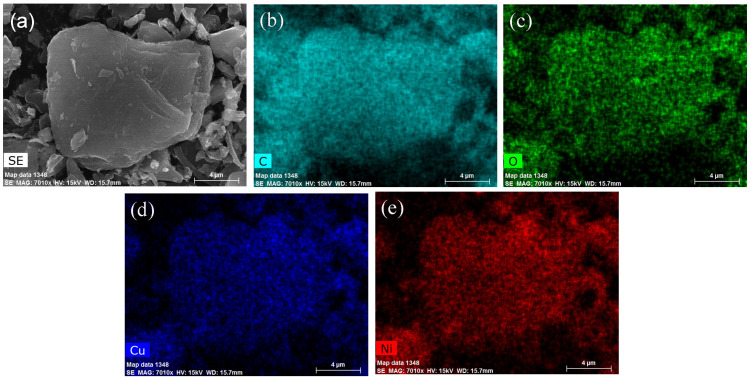
(**a**) SEM image of Ni_2_Cu_1_/RHPC. (**b**) C-mapped, (**c**) O-mapped, (**d**) Cu-mapped, and (**e**) Ni-mapped SEM-EDX images of Ni_2_Cu_1_/RHPC.

Additionally, the elemental mapping analysis, based on EDS, exhibited a homogeneous distribution of copper, nickel, and oxygen elements in the bimetallic nanocatalysts, respectively (Figure 5).

**Figure 6:**
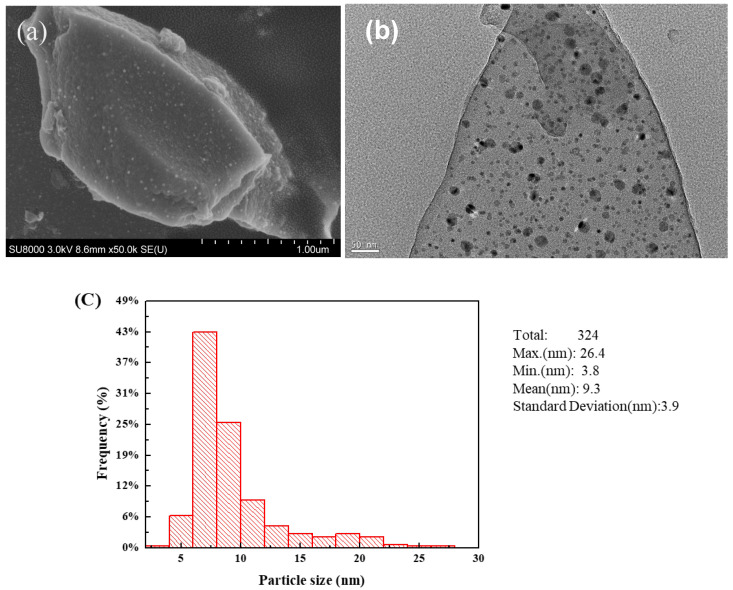
(**a**) SEM images, (**b**) TEM images, and (**c**) particle size distribution of Ni_2_Cu_1_/RHPC.

The SEM and TEM images of the Ni_2_Cu_1_/RHPC catalyst are shown in Figure 6. It can be seen from the SEM image (Figure 6a) and the TEM image (Figure 6b) that the NiCu bimetal is uniformly supported on the RHPC surface. Meanwhile, the particle size statistical results show that the average particle size of NiCu is 9.31 nm. This indicates that a small nano-sized NiCu alloy was synthesized. A 3 mg of sample was dispersed in 8 mL of ethanol solution and was sonicated before analysis. The sample was then dropped onto the surface of a double-layer copper mesh using a syringe and dried for testing. Particle size and lattice fringing analyses were performed using DigitalMicrograph 3.5 software (Figure 6c).

### 2.2. Catalytic Performance of Ni_x_Cu_y_/RHPC Catalysts

The selective hydrogenation of furfural was studied to evaluate the catalytic performance of the Ni_x_Cu_y_/RHPC catalysts. Table 3 shows the effect of the furfural selective hydrogenation performance of the Ni_2_Cu_1_/RHPC catalyst prepared by RHPC treated with different acidification conditions. It can be seen from the table that in the evaluation of the furfural hydrogenation performance, the selectivity of the Ni_2_Cu_1_/RHPC catalyst prepared without acid treatment of RHPC to tetrahydrofurfuryl alcohol was 93.4%, and the acid-prepared RHPC catalyst was used as the carrier. The selectivity of the Ni_2_Cu_1_/RHPC catalyst for tetrahydrofurfuryl alcohol is slightly higher. After comparing different acid concentration conditions, it can be seen that the Ni_2_Cu_1_/RHPC catalyst prepared by RHPC treated with 20% HNO_3_ has the highest selectivity to tetrahydrofurfuryl alcohol (selectivity of tetrahydrofurfuryl alcohol > 99%). This shows that the rich oxygen-containing functional groups on the surface of RHPC modified by nitric acid will affect the hydrogenation of FFA. Studies have found that among the various carbonyl functions, carboxyl groups play the most important role because they enhance the adsorption of furfuryl alcohol [17]. The acidic sites introduced by modifying porous carbon can activate the formation of furfuryloxy intermediates, which play a vital role in the formation of FOL and THFA [18]. When the acid concentration is further increased, the structural properties of the rice husk-based porous carbon will be severely damaged, which will affect the performance of the catalyst.

The effect of Ni_2_Cu_1_/RHPC, prepared at different temperatures, on the furfuralhydrogenation was also studied, as shown in Table 4. When the temperature increased from 300 °C to 500 °C, the conversion rate of furfural was 100%. The selectivity of THFA showed a tendency to increase first and then decrease. When the temperature was 400 °C, the highest selectivity of THFA was obtained. Thus, 400 °C was chosen as the optimum catalyst preparation temperature.

The effect of the Ni/Cu mole ratio of the catalyst on the catalytic performance of the furfural hydrogenation was shown in Table 5. Obviously, in the case of the monometallic Cu/RHPC catalyst, an extremely high selectivity of 100% to FOL was obtained, which revealed that Cu was responsible for the selective hydrogenation of C=O in furfural [29,30]. The furfural was completely converted, and FOL was further hydrogenated to THFA when Ni was added to the catalyst (Run 2). The highest selectivity to THFA was obtained by increasing the Ni/Cu molar ratio (Run 3). Unfortunately, the monometallic Ni/RHPC yielded a low selectivity of 26% to THFA (Run 4). This indicates that the high hydrogenation activity of the catalyst is not only due to the fact that it is nickel-rich, but also due to the interaction of NiCu. It is further explained that the interaction of NiCu is advantageous for the formation of THFA. It can be seen from Run 4 and Run 5 that the catalytic effect of Ni/RHPC and Cu/RHPC, after physical mixing, was lower than that of Ni_2_Cu_1_/RHPC. This indicates that the high catalytic performance of Ni_2_Cu_1_/RHPC is not simply the result of the mixture of Ni and Cu, but of a synergistic effect between Ni and Cu [11].

Effect of solvents on the catalytic activity of Ni_2_Cu_1_/RHPC is shown in Table 6. Initially, a series of slovents (Water, Ethanol and Decalin) were used to investigate the influence of chemical composition on the selectivity of furfural hydrogenantion. It was found that the highest selectivity of >99% to tetrahydrofurfuryl alcohol (THFA) derived from total hydrogenation of furfural when the water was used as solvent. When decalin and ethanol were used as solvents, furfural cannot be totally converted and furfuryl alcohol (FOL) can be obtained at the same time. Therefore, water was selected as the solvent in the reaction.

The effect of the reaction temperature on the catalytic activity of Ni_2_Cu_1_/RHPC for the selective hydrogenation of furfural is shown in Table 7. The results showed that the reaction temperature has a significant effect on the selectivity of THFA. The conversion of furfural is 92.4%, and the selectivity of 13.3% to THFA and 86.7% to FOL was obtained when the reaction temperature was 30 °C. This indicates that Ni_2_Cu_1_/RHAC can catalyze the hydrogenation of furfural to FOL and THFA under normal temperature conditions (the conversion of FFA is 92.4%). When the temperature was increased from 30 °C to 50 °C, furfural hydrogenation was further promoted. The optimum reaction temperature was 50 °C.

The effect of the furfural concentration on the catalytic performance of Ni_2_Cu_1_/RHPC for the selective hydrogenation of furfural was also studied, as shown in Table 8. When the furfural concentration was increased from 0.36 mmol to 1.20 mmol, the conversion of FFA was 100%, and the selectivity of THFA was remarkably lowered. When the furfural concentration was 1.20 mmol, the catalyst still showed higher activity. The selectivity of THFA was 85.3%, and the selectivity of FOL was 6.3%, with the production of a few by-products. Therefore, a suitable concentration of furfural was utilized to obtain a highly efficient catalytic hydrogenation product.

The recyclability of Ni_2_Cu_1_/RHAC was investigated along with the furfural hydrogenation reaction. The results are shown in Table 9. It could be seen that the selectivity of THFA was 96.7% when the catalyst was recycled five times. The catalyst could be regenerated after each run by activation in H_2_ for 2 h at 400 °C, and a high selectivity of THFA could be obtained. The catalyst still exhibits good activity and stability after being used five times. Our post-cycling ICP tests on the catalysts indicated that the reduction in catalyst loading after cycling may be the main reason for the reduction in catalytic reactions during cycling, with a reduction in elemental carryover (~30%) observed after the 7th cycle, but the furfural catalytic hydrogenation still maintained good conversions and selectivity, as shown in Table 9, from the results of the 5th cycle.

## 3. Experimental Section

### 3.1. Materials and Reagents

Ni(NO_3_)_2_·6H_2_O and Cu(NO_3_)_2_·3H_2_O were obtained from Sinopharm Chemical Reagent Co., Ltd. (Shanghai, China) (analytical reagent), and rice husk-based porous carbon (RHPC) was purchased from Jilin Kaiyu Biomass Development and Utilization Co., Ltd. (Jilin, China). Furfural analytical reagent was procured from Beijing Chemical Works.(Beijing, China) Before use, a small amount of anhydrous sodium carbonate was mixed in furfural for distillation under reduced pressure. A small quantity of the front cut fraction was discarded, and the main distillate fraction was collected. The distilled fractional distillation was wrapped in aluminum foil and stored at 0–4 °C.

### 3.2. Catalyst Preparation

RHPC was oxidized in a nitric acid solution at 120 °C for 4 h (acid concentrations of 10%, 20%, 30%, and 40%), and then RHPC was washed with a large amount of deionized water to achieve neutralization and then maintained in a vacuum drying box at 120 °C for 12 h. Various Ni_x_Cu_y_/RHPCs, with different Ni/Cu mole ratios (Ni/RHPC, Ni_2_Cu_1_/RHPC, Ni_1_Cu_2_/RHPC, Cu/RHPC), were fabricated using a wetness impregnation method. Ni(NO_3_)_2_·6H_2_O and Cu(NO_3_)_2_·3H_2_O, in different molar ratios, were dissolved in 8 mL of deionized water, before the addition of 0.5 g RHPC. Next, the mixture was exposed to ultrasound for 30 min, and then incubated at room temperature for 24 h, followed by drying at 110 °C for 12 h. Then, the catalyst was reduced in a H_2_ atmosphere at 400 °C for 2 h before reaction. Additionally, in order to investigate the effect of reduction temperature on catalytic performance, the bimetallic NiCu catalyst with an Ni/Cu mole ratio of 2:1 was reduced at different temperatures and marked as Ni_2_Cu_1_/RHPC-T (T = 300 °C, 350 °C, 400 °C, 450 °C, 500 °C).

### 3.3. Catalyst Characterization

Boehm titration was used to determine the surface acid content of RHPC. The Boehm titration method is a qualitative and quantitative analytical method based on the reactivity of acidic and basic surface oxides with different strengths. The carboxyl, lactone, and phenolic hydroxyl groups on the surface of activated carbon can be neutralized with bases of different strengths. Among these, the carboxyl groups were neutralized only by NaHCO_3_, while the carboxyl and lactone groups can be neutralized by Na_2_CO_3_, and the carboxyl, lactone, and phenolic hydroxyl groups can be neutralized by NaOH. The specific operation was as follows: three aliquots of 1 g acidified RHPC samples were measured into 100 mL conical flasks, and 25 mL of NaHCO_3_, Na_2_CO_3_, and NaOH solutions (calibrated concentration of 0.05 mol/L) were added; the filtrate was washed by filtration and collected after 24 h of continuous stirring on a multi-head magnetic stirrer. Then, the unreacted bases in the filtrate were titrated with a calibrated 0.05 mol/L HCl solution, using methyl red–bromocresol green as an indicator, respectively. Finally, the remaining amount of the added NaHCO_3_, Na_2_CO_3_ and NaOH solutions and the level of neutralization reaction with the surface functional groups were determined, based on the amount of standard HCl solution used, which in turn allowed for the calculation of the acidic functional group content. The consumption n (mmol/g) per unit mass of activated carbon surface acidic functional group content reacted with each standard alkaline solution was calculated according to Equations (1)–(6), as follows.

n_(NaOH)_ = [C_(NaOH)_ V_(NaOH_) − C_(HCl)_ V_(HCl)_]/m (1)

n_(Na_2_CO_3_)_ = [2C_(Na_2_CO_3_)_ V_(Na_2_CO_3_)_ − C_(HCl)_ V_(HCl)_]/m (2)

n_(NaHCO_3_)_ = [C_(NaHCO_3_)_ V_(NaHCO_3_)_ − C_(HCl)_ V_(HCl)_]/m (3)

where: V_(HCl)_ is the volume of standard hydrochloric acid solution used for titration (mL), and m is the mass of rice husk charcoal after acidification (g), so that the proportion of different acidic groups can be calculated.

n_(RCOOH)_ = n_(NaHCO_3_)_ (4)

n_(RCOOCOR)_ = n_(Na_2_CO_3_)_ − n_(NaHCO_3_)_ (5)

n_(ArOH)_ = n_(NaOH)_ − n_(Na_2_CO_3_)_ (6)

The FT-IR spectra were recorded in the wavenumber range of 4000–650 cm^−1^ using a Nicolet 6700 (Thermo Scientific, Waltham, MA, USA) spectrometer equipped with an MCT-A detector. The acid sites of various supports were qualitatively measured by the temperature programmed desorption (TPD) of ammonia. In the experiment, 30 mg of Ni_2_Cu_1_/RHPC was heated at 300 °C under He flow for 1 h. Then, it was saturated by a mixture of NH_3_ in He (60 mL/min) at 50 °C. The excess of ammonia was flushed out using He flow for 30 min. Ammonia desorption was carried out at a heating rate of 10 °C/min to 500 °C.

The X-ray powder diffraction (XRD) patterns were obtained on a B.V. Empyrean diffractometer with the settings of 40 kV and 40 mA using Cu Ka radiation, and data were collected at steps of 0.02° in the 2θ range of 10–80°. Scanning electron microscopy (SEM) images and elemental mapping data were collected with a Supra55 Zeiss (operating voltage 5 kV) equipped with an Oxford Instruments EDS X-ray spectrometer. Transmission electron microscopy (TEM) images were recorded on a TecnaiG2 S-Twin F20 instrument operated at 200 kV. XPS analysis was performed on an ESCALAB 250 X-ray photoelectron spectrometer (Thermo, USA) equipped with Al Kα_1,2_ monochromatized radiation using a 1486.6 eV X-ray source. Inductively coupled plasma optical emission spectroscopy (ICP-OES) analyses were performed on a Perkin Elmer OPTIMA 3300DV instrument to determine the Ni and Cu nanoparticle concentration in the Ni_x_Cu_y_/RHPC. N_2_ adsorption–desorption isotherms were measured by static N_2_ physisorption at −196 °C with an ASAP 2460 analyzer. The surface area was calculated using the multipoint Brunauer–Emmett–Teller (BET) method. The pore volume and pore size were calculated from desorption branches of the isotherms using the Barrett–Joyner–Halenda (BJH) method. The water contact angles of the Ni/RHPC were measured on a contact-angle system (8L-200B, KINO Scientific Instrument Inc., Boston, MA, USA), at room temperature.

### 3.4. Furfural Hydrogenation

The furfural hydrogenation reactions were carried out with various catalysts in a 100 mL stainless steel autoclave at a stirring speed of 500 rpm. For a typical run, 0.60 mmol of furfural, 30 mg of Ni_2_Cu_1_/RHPC catalyst, and 40 mL of water were introduced into the autoclave. After purging the reactor with pure H_2_ five times, the reactor was pressurized with pure H_2_ to 1 MPa and heated to 50 °C. After 1 h of reaction, the autoclave was cooled to room temperature, and the reaction solution was filtrated. The solid catalysts were filtered using a microporous filtering film (0.45 μm) and washed thoroughly three times with deionized water and ethanol. The reaction products were analyzed on a ShimadzuGC-2014C gas chromatograph equipped with a HP-Innowax column (30 m × 0.32 mm × 0.25 μm) and a flame ionization detector (FID), with nitrogen as the carrier gas.

The quantitative determination of the reaction products was carried out using the internal standard method. All results were evaluated on the basis of the amount of furfural obtained. The conversion of furfural (mol %), the selectivity (mol %) of the main products, and the carbon balance were calculated as:

Conversion = (1 − moles of furfural/moles of furfural loaded initially) × 100%

Selectivity of product = (moles of product/moles of furfural converted) × 100%

Carbon balance = (moles of products/moles of furfural converted) × 100%

## 4. Conclusions

In summary, we have developed NiCu alloy nanoparticle catalysts, with RHPC as the support. The characterization of the catalysts showed that the NiCu alloy nanoparticles uniformly covered the surface. The synergistic effect between NiCu bimetals is highly efficient for the catalytic hydrogenation of furfural. The modified RHPC surface with the acid sites and oxygen-containing functional groups can enhance the selectivity of THFA. An outstanding catalytic performance of Ni_2_Cu_1_/RHPC in the conversion of furfural to THFA was achieved, with a 100% conversion of furfural and a 98.9% yield of THFA, under mild reaction conditions (50 °C, 1 MPa, 1 h, 500 r/min). Moreover, Ni_2_Cu_1_/RHPC also exhibits good stability in the recycle tests. The efficient CuNi alloy nanocatalysts are not only promising candidates for the effective upgrading of biomass-derived furfural, but also provide useful guidance for the rational design of non-noble bimetallic nanocatalysts for hydrogenative transformations.

## Figures and Tables

**Table 1:** The composition of Ni and Cu in the Ni_x_Cu_y_/RHPC catalysts ^a^.

Run	Catalyst	wt %	
		Ni	Cu
1	Cu/RHPC	0	21.4
2	Ni_1_Cu_2_/RHPC	7.0	15.7
3	Ni_2_Cu_1_/RHPC	15.8	8.3
4	Ni/RHPC	22.0	0
5	Ni_2_Cu_1_/RHAC-Recycled	9.4	5.0

^a^ Determined by ICP-AES.

**Table 2:** The pore structure parameters of RHPC and Ni_2_Cu_1_/RHPC catalysts, before and after nitric acid treatment.

Samples	S_BET_ m^2^/g	V_total_ cm^3^/g	V_micro_ cm^3^/g	V_meso_ cm^3^/g	V_micro_/V_total_ %	V_meso_/V_total_ %	D_ave._ nm
RHPC	1847	1.06	0.65	0.41	61	39	2.3
RHPC-HNO_3_ ^a^	974	0.60	0.26	0.34	43	57	1.8
Ni_2_Cu_1_/RHPC	591	0.37	0.12	0.25	32	68	2.5

^a^ Treated with 20% HNO_3_ at 120 °C for 4 h.

**Table 3:** Effects of acidification conditions of the RHPC support on the catalytic activity of Ni_2_Cu_1_/RHPC catalysts for the hydrogenation of furfural ^a^.

Run	Acidification Conditions	Con./%	Sel./%	
			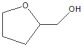	Others
1	—	>99.9	93	7
2	10% HNO_3_ 25 °C	>99.9	98	2
3	10% HNO_3_ 120 °C	>99.9	98	2
4	20% HNO_3_ 120 °C	>99.9	>99	0
5	30% HNO_3_ 120 °C	>99.9	97	3
6	40% HNO_3_ 120 °C	>99.9	97	3

^a^ Reaction conditions: 24 wt% Ni_2_Cu_1_/RHPC (30 mg), furfural (0.60 mmol), H_2_O (40 mL), *p* (H_2_) = 1 MPa, 50 °C, 1 h, 500 r/min.

**Table 4:** Effect of reduction temperature on the catalytic activity of Ni_2_Cu_1_/RHPC for the hydrogenation of furfural ^a^.

Run	T/°C	Con./%	Sel./%	
			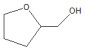	Others
1	300	>99.9	94	6
2	350	>99.9	96	4
3	400	>99.9	>99	0
4	450	>99.9	98	2
5	500	>99.9	96	4

^a^ Reaction conditions: 24 wt% Ni_2_Cu_1_/RHPC (30 mg), furfural (0.60 mmol), H_2_O (40 mL), *p* (H_2_) = 1.0 MPa, 50 °C, 1 h, 500 r/min.

**Table 5:** Effect of the Ni/Cu ratio of the catalyst on the catalytic performance for the hydrogenation of furfural.

Run	Catalyst	Con./%	Sel./%		
			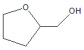	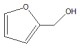	Others
1 ^a^	Cu/RHPC	63	0	100	0
2 ^a^	Ni_1_Cu_2_/RHPC	>99.9	74	21	5
3 ^a^	Ni_2_Cu_1_/RHPC	>99.9	>99	0	0
4 ^a^	Ni/RHPC	98	26	67	7
5 ^b^	Ni/RHPC (20 mg)+ Cu/RHPC (10 mg)	>99.9	86	9	5

^a^ Reaction conditions: Ni_x_Cu_y_/RHPC (30 mg), Furfural (0.60 mmol), H_2_O (40 mL), *p* (H_2_) = 1.0 MPa, 50 °C,1 h, 500 r/min. ^b^ Reaction conditions: Cat. (30 mg), Furfural (0.60 mmol), H_2_O (40 mL), *p* (H_2_) = 1.0 MPa, 50 °C, 1 h, 500 r/min.

**Table 6:** Effect of solvents on the catalytic activity of Ni_2_Cu_1_/RHPC for the hydrogenation of furfural ^a^.

Run	Solvent	Con./%	Sel./%	
			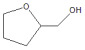	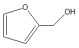
1	Water	>99.9	>99	0
2	Ethanol	99	31	69
3	Decalin	86	29	71

^a^ Reaction conditions: 24 wt% Ni_2_Cu_1_/RHPC (30 mg), furfural (0.36 mmol), solvent (40 mL), *p* (H_2_) = 1.0 MPa, 50 °C, 1 h, 500 r/min.

**Table 7:** Effect of reaction temperature on the catalytic activity of Ni_2_Cu_1_/RHPC for the hydrogenation of furfural ^a^.

Run	T/°C	Con./%	Sel./%		
			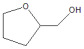	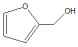	Others
1	30	92	13	87	0
2	40	>99.9	54	41	5
3	50	>99.9	>99	0	0

^a^ Reaction conditions: 24 wt% Ni_2_Cu_1_/RHPC (30 mg), furfural (0.60 mmol), H_2_O (40 mL), *p* (H_2_) = 1.0 MPa, 1 h, 500 r/min.

**Table 8:** Effect of the amount of furfural on the catalytic performance of Ni_2_Cu_1_/RHPC for the hydrogenation of furfural ^a^.

Run	n_furfural_/mmol	Con./%	Sel./%		
			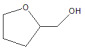	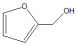	Others
1	0.36	>99.9	>99	0	0
2	0.60	>99.9	>99	0	0
3	0.90	>99.9	97	0	3
4	1.20	>99.9	85	7	8

^a^ Reaction conditions: 24 wt% Ni_2_Cu_1_/RHPC (30 mg), H_2_O (40 mL), *p* (H_2_) = 1.0 MPa, 50 °C, 1 h, 500 r/min.

**Table 9:** Recyclability of the Ni_2_Cu_1_/RHPC catalyst for the hydrogenation of furfural ^a^.

Run	Cycle	Con./%	Sel./%	
			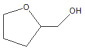	Others
1	—	>99.9	>99	0
2	1st	>99.9	99	1
3	2nd	>99.9	97	3
4	3rd	>99.9	97	3
5	4th	>99.9	97	3
6	5th	>99.9	97	3

^a^ Reaction conditions: 24 wt% Ni_2_Cu_1_/RHPC (30 mg), furfural (0.60 mmol), H_2_O (40 mL), *p* (H_2_) = 1 MPa, 50 °C, 1 h, 500 r/min.

## Data Availability

Data are contained within the article.
